# Clinical impact of reducing the frequency of clozapine monitoring: controlled mirror-image cohort study

**DOI:** 10.1192/bjp.2023.44

**Published:** 2023-08

**Authors:** Ebenezer Oloyede, Olubanke Dzahini, Zadro Abolou, Siobhan Gee, Eromona Whiskey, Diksha Malhotra, Masuma Hussain, Ian Osborne, Cecilia Casetta, Philip McGuire, James H. MacCabe, David Taylor

**Affiliations:** South London and Maudsley NHS Foundation Trust, London, UK; and Department of Psychiatry, University of Oxford, Warneford Hospital, Oxford, UK; South London and Maudsley NHS Foundation Trust, London, UK; and Institute of Pharmaceutical Science, King's College London, London, UK; Barnet, Enfield and Haringey Mental Health Trust, London, UK; East London NHS Foundation Trust, London, UK; South West London and St George's Mental Health NHS Trust, London, UK; South London and Maudsley NHS Foundation Trust, London, UK; and Institute of Psychiatry, Psychology & Neuroscience, King's College London, London, UK

**Keywords:** Clozapine, COVID-19, agranulocytosis, neutropenia, pharmacovigilance

## Abstract

**Background:**

To minimise infection during COVID-19, the clozapine haematological monitoring interval was extended from 4-weekly to 12-weekly intervals in South London and Maudsley NHS Foundation Trust.

**Aims:**

To investigate the impact of this temporary policy change on clinical and safety outcomes.

**Method:**

All patients who received clozapine treatment with extended (12-weekly) monitoring in a large London National Health Service trust were included in a 1-year mirror-image study. A comparison group was selected with standard monitoring. The proportion of participants with mild to severe neutropenia and the proportion of participants attending the emergency department for clozapine-induced severe neutropenia treatment during the follow-up period were compared. Psychiatric hospital admission rates, clozapine dose and concomitant psychotropic medication in the 1 year before and the 1 year after extended monitoring were compared. All-cause clozapine discontinuation at 1-year follow-up was examined.

**Results:**

Of 569 participants, 459 received clozapine with extended monitoring and 110 controls continued as normal. The total person-years were 458 in the intervention group and 109 in the control group, with a median follow-up time of 1 year in both groups. During follow-up, two participants (0.4%) recorded mild to moderate neutropenia in the intervention group and one (0.9%) in the control group. There was no difference in the incidence of haematological events between the two groups (IRR = 0.48, 95% CI 0.02–28.15, *P* = 0.29). All neutropenia cases in the intervention group were mild, co-occurring during COVID-19 infection. The median number of admissions per patient during the pre-mirror period remained unchanged (0, IQR = 0) during the post-mirror period. There was one death in the control group, secondary to COVID-19 infection.

**Conclusions:**

There was no evidence that the incidence of severe neutropenia was increased in those receiving extended monitoring.

Clozapine has been regarded as the gold-standard antipsychotic for treatment-resistant psychosis since the seminal study of Kane and colleagues over three decades ago.^1^ Despite its strong evidence base, clozapine remains grossly under-prescribed in clinical practice.^2^ Although reasons for this are multifaceted, a commonly cited influence is the need for mandatory haematological monitoring.^3^ This monitoring practice is intended to mitigate against the rare but potentially fatal risk of agranulocytosis (reported in 0.4% of patients)^4^ associated with clozapine treatment.^2^

In the UK and many other countries, the frequency of haematological monitoring during clozapine treatment reduces to 4-weekly intervals after 1 year of monitoring at weekly and 2-weekly intervals, and monitoring must be continued until the patient discontinues clozapine.^2^ During the COVID-19 pandemic, local guideline changes temporarily permitted patients who were deemed at low risk to have their full blood count (FBC) monitoring extended from 4-weekly to 12-weekly intervals to reduce the risk of exposure to the virus (Fig. 1).^5^ This decision was supported by several lines of evidence demonstrating the risk of agranulocytosis to be very low after 1 year of treatment.^6,7^ Similar recommendations were made by the US Food and Drug Administration, allowing for the relaxation of the Clozapine Risk Evaluation and Mitigation Strategies (Clozapine REMS) such that clozapine monitoring could be based on clinical judgement, i.e. whether the benefits of deferring monitoring outweighed the risk of continuing treatment without an updated absolute neutrophil count (ANC).^8^ The importance of these recommendations has since been emphasised by recent evidence associating clozapine use with an increased risk of COVID-19 infection which, in some cases, results in clozapine intoxication.^9^

**Fig. 1:**
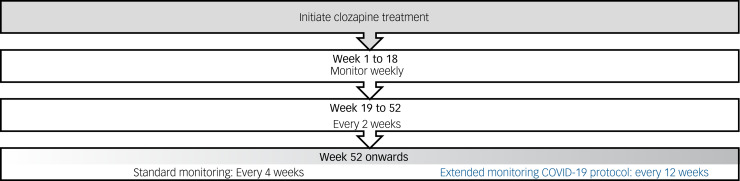
Summary of the UK Medicines & Healthcare products Regulatory Agency (MHRA) guidelines for clozapine monitoring frequency (white cell count and absolute neutrophil count) and the emergency 12-weekly monitoring protocol.

Since then, there have been calls for extended monitoring to be implemented in routine practice to help ease restrictions to clozapine use. At present, it is unknown how this policy change influences clinical outcomes in those receiving clozapine treatment. In this study, our objective was to explore the impact of this policy change on clinical and safety outcomes, including mild to severe neutropenia, hospital admission rates and adherence to treatment. Our hypothesis was that extended monitoring would not adversely affect clinical and safety outcomes in those receiving clozapine treatment.

## Method

### Data source and study sample

We examined the clozapine monitoring service database Zaponex Treatment Access System (ZTAS), operated by Leyden Delta BV, for patients registered for clozapine use by South London and Maudsley (SLaM) NHS Foundation Trust and East London NHS Foundation Trust (ELFT). SLaM provides mental health services to 1.2 million people across four south London boroughs and ELFT serves a population of 0.8 million across east London. In both trusts, out-patients access haematological monitoring in a community-based clozapine clinic, where venous samples are analysed using a point-of-care device. Patients’ haematological data were collected from ZTAS. Patient demographics and clinical data, such as the duration of illness, number of psychiatric admissions, ethnicity and the date of clozapine initiation, were collected from electronic medical records. Pharmacy dispensing records were used to collect medication data. We excluded those in forensic mental health services from the mirror-image analysis, specifically because these patients had legal barriers to discharge independent of mental state. The comparison group comprised patients from ELFT who were registered for extended monitoring but who ultimately received routine monitoring as the extended monitoring policy was not introduced. All procedures involving human subjects/patients were approved by each trust's Drug and Therapeutics Committee (DTC/2021/30), the locally designated approval committee for all non-interventional prescribing outcome evaluations, and the analysis used anonymised clinical data.

Strengthening the Reporting of Observational studies in Epidemiology (STROBE) reporting guidelines were followed (Supplementary Material, available at https://dx.doi.org/10.1192/bjp.2023.44).

### Study design

**Fig. 2:**
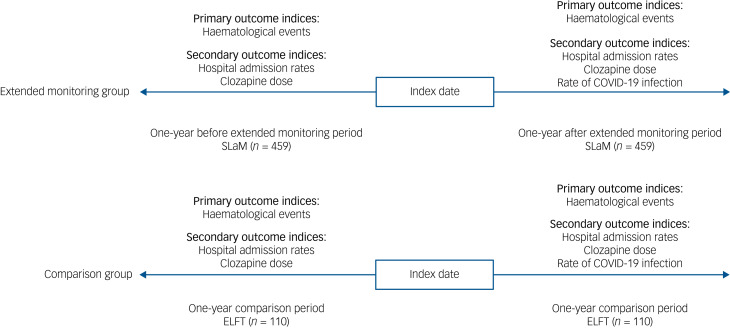
The mirror-image design. SLaM, South London and Maudsley NHS Foundation Trust; ELFT, East London NHS Foundation Trust.

All patients registered for extended monitoring in SLaM and ELFT between April 2020 and July 2021 were included in the study. Participants did not have to remain on clozapine treatment throughout to be included in the study. Eligibility criteria for extended monitoring agreed by expert consensus included having an absolute neutrophil count (ANC) ≥2.0 × 10^9^/L (or ≥1.5 × 10^9^/L if there was a history of benign ethnic neutropenia) and demonstrated adherence to clozapine for at least 12 months.^5^ This change was outside the terms of the product licence and required notification to the relevant clozapine monitoring service, ZTAS. All participants were based in the community setting at baseline. As primary outcomes, the proportion of participants with mild to severe neutropenia and the proportion of participants who attended emergency departments for clozapine-induced severe neutropenia treatment (i.e. agranulocytosis) during the follow-up period were compared between those receiving extended monitoring and standard monitoring. Mild to moderate neutropenia was defined as an ANC ≥0.5 and <1.5 × 10^9^/L; severe neutropenia defined as an ANC <0.5 × 10^9^/L.^2^ Mild to severe neutropenia was selected because an ANC <0.5 × 10^9^/L requires patients to stop clozapine in the UK.^2^ The study design is summarised in Fig. 2.

For the mirror-image analysis, psychiatric hospital admission rate, change in clozapine dose and concomitant psychotropic medication were compared 1 year before and after the index date. The index date or mirror point was defined as the last blood test date before haematological monitoring was changed from 4-weekly to 12-weekly intervals in both cohorts.

The proportion of participants admitted to psychiatric hospital and the mean number of admissions were collected and compared in the 1 year before and after the index date. The reasons for hospital admission were recorded. We did not investigate bed-days as they may be affected by factors outside clinical status, such as accommodation availability.

Participants were followed up for 1 year after the index date and all-cause treatment discontinuation was recorded, including the date and reason. Treatment discontinuation was defined as discontinuation for longer than 7 consecutive days. Where applicable, the date and reason for reverting to standard monitoring were obtained from descriptive medical records. Infection with COVID-19 during the follow-up period was examined. We used ZTAS data to determine whether there was a haematological event during COVID-19 infection.

### Statistical analysis

Baseline patient demographic and medication characteristics were summarised using descriptive statistics. Kolmogorov–Smirnov normality tests for continuous outcome variables were performed. Means and standard deviations (s.d.) were calculated for continuous data and frequencies and percentages were calculated for categorical data. Incidence rates were calculated for the intervention and comparison groups as the number of cases divided by the number of person-years. Person-years were defined as the years contributed by each participant from the index date to the first haematological event, treatment discontinuation or end of follow-up. The incidence rate ratio (IRR) was calculated for the first haematological event in the follow-up period. Risk ratios (RR) were calculated for hospital admission, treatment discontinuation and infection with COVID-19 in the follow-up period. Regression analysis was planned for admission data but not performed owing to low event rates and lack of clinically meaningful differences – instead, unadjusted effect estimates were reported. Background risks of mild to moderate neutropenia and of agranulocytosis after 1 year were reported to be 0.7% and 0.07% respectively in Atkin et al (1996) in a UK and Ireland database study.^7^ The number of participants required in the intervention group to provide 95% power is 429 to detect one case of neutropenia and 4286 to detect one case of agranulocytosis. Statistical significance was considered demonstrated if the *P*-value was less than 0.05. Statistical analysis was performed using R Studio 2020 for Windows (RStudio PBC, Boston, USA; https://posit.co/products/open-source/rstudio/).

### Role of the funding source

There was no funding source for this study. The corresponding author had full access to all the data in the study and had final responsibility for the decision to submit for publication.

## Results

### Patient characteristics

A total of 569 patients were included in this study. Of these, 459 were receiving clozapine treatment with extended monitoring at data collection (Fig. 2). The total person-years for the intervention group were 458 and the median follow-up time was 1 year. The mean age of the intervention cohort was 49 years (s.d. = 12), 67% were male and the mean duration of clozapine use was 11 years (s.d. = 6). A total of 110 comparison participants were included in this study. The total person-years for the comparison group were 109 and the median follow-up time was 1 year. The mean age of the comparison group was 48 years (s.d. = 10), 66% were male and the mean duration of clozapine use was 14 years (s.d. = 7). Clinical and demographic characteristics of the study population are shown in Table 1.

**Table 1:** Baseline sociodemographic and clinical characteristics of participants in the extended monitoring group compared with the control group

Baseline characteristics	Extended monitoring group (*n* = 459)	Control group (*n* = 110)
Male gender, *n* (%)	308 (67)	73 (66)
Age, years: mean (s.d.)	49 (12)	48 (10)
Duration of illness, years: mean (s.d.)	19 (6)	25 (16)
Clozapine dose, mg: mean (s.d.)	356 (146)	366 (153)
BEN diagnosis, *n* (%)	29 (6)	0 (0)
Time to clozapine initiation from illness onset, years		
Mean (s.d.)	8 (7)	8 (6)
Median (IQR)	5 (3–12)	7 (2–12)
Duration of clozapine treatment, years		
Mean (s.d.)	11 (6)	14 (7)
Median (IQR)	13 (6–17)	16 (11–19)
ICD-10 diagnosis, *n* (%)		
F20 Paranoid schizophrenia	354 (77)	102 (93)
F25 Schizoaffective disorder	59 (13)	4 (3)
F31 Bipolar disorder	12 (3)	0 (0)
Other	34 (8)	4 (0)
Psychiatric comorbidity, *n* (%)	48 (10)	16 (15)
Ethnicity, *n* (%)		
White	193 (42)	57 (52)
Black	164 (36)	37 (34)
Asian	40 (9)	10 (9)
Other	62 (13)	6 (5)
Concomitant psychotropic medication, *n* (%)		
Antipsychotic	126 (27)	34 (31)
Mood stabiliser^a^	146 (32)	23 (21)
Antidepressant	135 (29)	26 (24)

BEN, benign ethnic neutropenia; IQR, interquartile range.

a. Lithium, valproate or lamotrigine.

### Haematological events

Two of the 459 (0.4%) participants receiving extended monitoring had a total of 10 episodes of mild to moderate neutropenia during the post-mirror period; there were no cases of severe neutropenia. One of the 110 comparison group participants (0.9%) recorded 1 instance of mild to moderate neutropenia during the post-mirror period; there were no cases of severe neutropenia. All cases of neutropenia in the intervention group occurred during infection with COVID-19. There were no acute admissions for treatment of clozapine-induced severe neutropenia in the intervention or comparison groups during the post-mirror period.

There was an incidence of 4 haematological events per 1000 person-years in the intervention group, compared with 9 haematological events per 1000 person-years in the comparison group. The IRR of haematological events for participants with extended monitoring compared with standard monitoring was 0.48 (95% CI 0.02–28.15, *P* = 0.29).

### Continuation with clozapine treatment and extended monitoring

At 1-year follow-up, 457 participants (99.6%) remained on clozapine in the intervention group and 109 participants (99.1%) remained on clozapine in the comparison group. The RR for treatment discontinuation with extended monitoring compared with standard monitoring was 0.48 (95% CI 0.04–5.23, *P* = 0.54). The reason for discontinuation in the intervention group was patient request (*n* = 2). The reason for discontinuation in the comparison group was death secondary to infection with COVID-19 (*n* = 1). Among participants who were receiving extended monitoring, two (0.4%) were reverted to standard monitoring. Reasons for reverting to extended monitoring were clozapine interruption secondary to infection with COVID-19 (*n* = 1) and clozapine interruption after not attending blood tests (*n* = 1).

### Admissions and medication

Of the 459 patients in the intervention group, 23 (5%) were admitted to psychiatric hospital during the pre-mirror period and 10 (2%) during the post-mirror period. There was no change in the median number of psychiatric admissions in the year after extended monitoring compared with the year before in the intervention group (0, IQR = 0 versus 0, IQR = 0).

Of the 110 patients in the comparison group, 9 (8%) were admitted to psychiatric in-patient care during the pre-mirror period and 5 (5%) during the post-mirror period. The median number of admissions per patient during the pre-mirror and the post-mirror periods remained unchanged, at 0 (IQR = 0).

In the intervention group, 52% (12 of 23) of admissions during the pre-mirror period were due to non-adherence to clozapine treatment. During the post-mirror period, 60% (6 of 10) of admissions were due to non-adherence to clozapine treatment. In the comparison group, 55% (5 of 9) of admissions during the pre-mirror period were due to non-adherence with clozapine. During the post-mirror period, 60% (3 of 5) of admissions were due to non-adherence to clozapine. The RR for hospital admission during the follow-up period with extended monitoring compared with standard monitoring was 0.5 (95% CI 0.17–1.37, *P* = 0.17).

The median clozapine dose was unchanged from the pre- to post-mirror period in the intervention group (325 mg, IQR = 175). In the intervention group, the proportion of participants prescribed concomitant antipsychotics increased from 126 (27%) to 133 (29%); the proportion prescribed mood stabilisers increased from 146 (32%) to 151 (33%) and the proportion prescribed antidepressants reduced from 135 (29%) to 130 (28%).

In the comparison group, there was no change in the median clozapine dose in the pre- to post-mirror period (350 mg, IQR = 200 versus 350 mg, IQR = 162.5). In the comparison group, the proportion of participants prescribed concomitant antipsychotics increased from 34 (31%) to 35 (32%); the proportion prescribed mood stabilisers increased from 23 (21%) to 27 (25%) and the proportion prescribed antidepressants reduced from 26 (24%) to 22 (20%).

### COVID-19 infection

Among the 459 individuals receiving extended monitoring, 25 (5.5%) tested positive for infection with COVID-19 during the follow-up period. In the comparison group of 110 individuals, 7 (6.4%) tested positive for infection with COVID-19 during the follow-up period. The RR for infection with COVID-19 with extended monitoring compared with standard monitoring was 0.86 (95% CI 0.38–1.93, *P* = 0.70). These individuals did not have to revert to 4-weekly monitoring but may have been subject to increased monitoring if ANC results were below threshold.

## Discussion

### Our findings

The results of our study indicated that extending the haematological monitoring interval from 4-weekly to 12-weekly did not increase the incidence of life-threatening agranulocytosis in people taking clozapine. Our findings provide additional evidence to support existing calls for extended monitoring to be adopted in routine practice, to help address the prevalent under-utilisation of clozapine.^6,7,10,11^

### Comparison with other studies

The safety of extended monitoring has been previously shown by several studies.^6,10,12,13^ To our knowledge, this is the first and largest study to investigate the clinical impact of extended monitoring in the UK. During the pandemic, data from a group of 50 patients (27%) in the USA demonstrated no clinical decompensation during a 6-week observational period after receiving extended monitoring.^8^ Similarly, a retrospective study in Qatar described the absence of neutropenia and fewer admissions to psychiatric and medical services in those receiving extended monitoring.^14^ Overall, these findings suggest that a significant proportion of patients can safely receive clozapine with extended monitoring. Although some observers have expressed concerns about reducing haematological monitoring for those receiving clozapine treatment,^15^ predominantly because of the risk of late-onset neutropenia, these events remain rare and comparable to other medications that are not subject to the same monitoring scrutiny.^16^ Indeed, initial guidelines for the psychotropic carbamazepine extensively advocated routine monitoring for blood dyscrasias, but this was eventually removed owing to the rarity of the adverse effect.^17^

### Haematological and clinical outcomes

In our study there was no increase in people admitted with life-threatening haematological adverse events related to clozapine use in the group who received extended monitoring. Although incidence rates were higher than reported in previous studies, all cases of mild neutropenia were likely related to infection with COVID-19.^9^ This is consistent with evidence that the risks of neutropenia and agranulocytosis are very low after 1 year of treatment.^6,10^ In fact, more recent data, including meta-analyses, have challenged the notion that clozapine poses an increased risk of neutropenia compared with conventional antipsychotics.^18,19^ Of note, in the intervention group, a higher proportion had identified benign ethnic neutropenia compared with the comparison group, which might have increased the likelihood of detecting mild to moderate neutropenia in the former cohort. Moreover, when compared with larger historical data our extended monitoring cohort had similar haematological outcomes. In a total of 21 473 patients, Kumar^20^ reported a neutropenia incidence of 7.43 per 1000 patient-years. Although definitions of moderate to severe neutropenia were marginally different between studies, when calculated with Kumar as a control, the IRR is 0.59 (95% CI: 0.12–2.25, *P* = 0.36), compared with 0.48 (95% CI 0.02–28.15, *P* = 0.29) with our control group.

In our study, we found no difference in psychiatric hospital admission rates in those receiving extended monitoring and the comparison group. In addition, clozapine doses and concomitant medications remained unchanged during the observation period, suggesting relative stability of mental state despite reduced face-to-face service contact. Notably, previous studies have suggested that some of the superior clinical benefits with clozapine, such as reduced mortality and self-harm compared with other antipsychotics, are independent of the increased service utilisation and clinician contact.^21,22^ Moreover, there was no substantial difference in clozapine non-adherence between the two groups despite fewer contacts. Nevertheless, it is also plausible that the absence of group differences in our study was due to the relatively short follow-up.

### COVID-19 infection

Undoubtedly, it is challenging to disentangle the effects of the clozapine policy changes from those of the COVID-19 pandemic, including social distancing and the resultant reduction in face-to-face contacts with mental health services. Nevertheless, we attempted to do this in our study through a mirror-image design, with a control group receiving care as usual. Contrary to expectations, we found that 5% of participants receiving extended monitoring contracted COVID-19, compared with 6% of those receiving treatment as usual, suggesting that the reduced contact was not effective in achieving the intended prevention or reduction of COVID-19 infection. Nevertheless, these data provide justification for the policy change to improve service utilisation, as previous studies have shown stringent clozapine monitoring to be a significant barrier to clozapine utilisation.^3^ Moreover, it could be argued that this intervention may contribute to improved patient satisfaction.^3^ Although COVID-19 has undoubtedly had a negative impact on clinical care in many areas, this policy change appears to provide a real opportunity to implement changes to improve clozapine utilisation rates in the UK and possibly beyond.

### Clinical implications

There are wide international disparities between guidelines on the monitoring of clozapine treatment and many of the differences in recommendations appear arbitrary in nature. From a scientific standpoint, there has been much debate long before the pandemic regarding the necessity of haematological monitoring of clozapine in perpetuity, particularly regarding its indefinite nature and thresholds for discontinuation.^23^ Cases of clozapine-related agranulocytosis usually occur in the first 6 months of treatment,^7^ which suggests that this adverse drug reaction can be identified in the early stages by standard monitoring. In addition to this, there is accumulating evidence questioning the need for haematological monitoring beyond the first 6–12 months of clozapine treatment.^10,18,19^ In fact, authors have noted that the risk of neutropenia is comparable to that with other psychotropics not subject to the same blood testing requirements and that the stringency of current practice is a result of isolated cases early in clozapine's development. The impact of these early cases has distorted the perception of clozapine's relative risks, a perception that continues to dominate its widely reported clinical benefits for seriously and persistently ill patients, especially considering the limited alternative treatment strategies to address inadequate antipsychotic response. Coupled with this is the recently gained awareness that twice-monthly or monthly monitoring will have a low likelihood of capturing the characteristic rapid drop in neutrophil granulocytes during clozapine-induced agranulocytosis. Instead, a more pragmatic strategy of intensive haematological monitoring in the first year of clozapine initiation followed by selective haematological monitoring in case of febrile illnesses or pharyngitis needs to be explored. In their commendable review, over 10 years ago the Netherlands Clozapine Collaboration Group recommended lowering the frequency of blood tests to four times a year (off-label) for mentally competent and adequately informed patients who request it.^23^ As previously demonstrated, stringent monitoring of people receiving clozapine treatment imposes an unnecessary burden on them.^3^ Such monitoring also has important economic implications,^24^ contributing to inequalities in access to healthcare while providing negligible benefit. It is our opinion that the relaxation of clozapine blood monitoring requirements prompted by the pandemic provides an opportunity for revising the protocol to be followed once the public health emergency has resolved.

### Strengths, limitations and future studies

This study has several strengths. Our study's within-participant design helped to minimise the impact of individual-level confounders on the number of in-patient admissions by effectively comparing patients against themselves. Furthermore, inclusion of a control group separates the impact of the monitoring from that of the pandemic itself. However, a key limitation of the study is that patients who received extended monitoring were selected based on an absence of prior haematological events and demonstrated adherence to clozapine. This limits the generalisability of our results because participants were selected who were deemed at low risk of complications with extended monitoring. Nevertheless, as mentioned above, the utility of monthly monitoring has been questioned from a clinical and economic perspective. A further limitation is that our study includes a relatively short follow-up period. Future data may be required to determine clinical impact on a long-term basis. However, it has been demonstrated in one of our care settings that most patients will continue clozapine treatment long term and of those who do discontinue treatment, most do so within the first 4 years.^25^ As most of our patients were established on clozapine long before extended monitoring was introduced, it is plausible that discontinuation rates will remain low if extended monitoring is implemented in similar populations. Although our findings are encouraging, it should be noted that our sample is small considering that true clozapine-induced severe neutropenia is a rare adverse drug reaction. Our sample size of 459 participants in the extended monitoring group provides a power of 96% to detect one case of neutropenia and 27% to detect agranulocytosis. Despite insufficient power to detect agranulocytosis, we are still able to estimate the upper limit of the 95% CI for agranulocytosis risk. Applying the rule of three to estimate the upper limit of the 95% CI of agranulocytosis risk under the extended monitoring scheme, we can be 95% confident that the maximum risk does not exceed 0.6%, i.e. the 95% CI is 0–0.6%.^26^ Moreover, from a clinical perspective, it is plausible that a larger sample would have resulted in more non-clozapine induced, transient cases of neutropenia in the standard monitoring group compared with the extended monitoring group, simply due to the increased frequency of monitoring. It is unknown whether a reduction in the amount of physical contact patients had with clinicians was compensated by increased contact by telephone or other means, so the amount of monitoring patients receive might have been compromised. Finally, our study is underpowered to identify potential differences in haematological events. Moreover, the different monitoring between intervention and control groups makes comparison of haematological outcomes very difficult to interpret. Despite these limitations, this is the largest study to investigate the effectiveness of extended monitoring in patients receiving clozapine treatment. Future larger studies are required to confirm our preliminary findings.

## Data Availability

The data that support the findings of this study are available from the corresponding author (E.O.) on reasonable request.

## Supplementary material

For supplementary material accompanying this paper visit https://doi.org/10.1192/bjp.2023.44. click here to view supplementary material
